# HbA_1c_ variability is independently associated with progression of diabetic kidney disease in an urban multi-ethnic cohort of people with type 1 diabetes

**DOI:** 10.1007/s00125-024-06197-2

**Published:** 2024-06-20

**Authors:** Ananya Muthukumar, Layla Badawy, Anastasios Mangelis, Prashant Vas, Stephen Thomas, Aicha Gouber, Salma Ayis, Janaka Karalliedde

**Affiliations:** 1 Faculty of Life Sciences and Medicine, King’s College London, London, UK; 2 Department of Diabetes and Endocrinology, Guy’s and St Thomas’ NHS Foundation Trust, London, UK

**Keywords:** African Caribbean, Diabetic nephropathy, eGFR, Ethnicity, Glycaemic variability, HbA_1c_ variability, Type 1 diabetes

## Abstract

**Aims/hypothesis:**

The role of HbA_1c_ variability in the progression of diabetic kidney disease is unclear, with most studies to date performed in White populations and limited data on its role in predicting advanced kidney outcomes. Our aim was to evaluate if long-term intra-individual HbA_1c_ variability is a risk factor for kidney disease progression (defined as an eGFR decline of ≥50% from baseline with a final eGFR of <30 ml/min per 1.73 m^2^) in an ethnically heterogeneous cohort of people with type 1 diabetes with a preserved eGFR ≥45 ml/min per 1.73 m^2^ at baseline.

**Methods:**

Electronic health record data from people attending outpatient clinics between 2004 and 2018 in two large university hospitals in London were collected. HbA_1c_ variability was assessed using three distinct methods: (1) SD of HbA_1c_ (SD-HbA_1c_); (2) visit-adjusted SD (adj-HbA_1c_): SD-HbA_1c_/√*n*/(*n*–1), where *n* is the number of HbA_1c_ measurements per participant; and (3) CV (CV-HbA_1c_): SD-HbA_1c_/mean-HbA_1c_. All participants had six or more follow-up HbA_1c_ measurements. The eGFR was measured using the Chronic Kidney Disease Epidemiology Collaboration equation and clinical/biochemical results from routine care were extracted from electronic health records.

**Results:**

In total, 3466 participants (50% female, 78% White, 13% African Caribbean, 3% Asian and 6% of mixed heritage or self-reporting as ‘other’) were followed for a median (IQR) of 8.2 (4.2–11.6) years. Of this cohort, 249 (7%) showed kidney disease progression. Higher HbA_1c_ variability was independently associated with a higher risk of kidney disease progression, with HRs (95% CIs) of 7.76 (4.54, 13.26), 2.62 (1.75, 3.94) and 5.46 (3.40, 8.79) (lowest vs highest HbA_1c_ variability quartile) for methods 1–3, respectively. Increasing age, baseline HbA_1c_, systolic BP and urinary albumin/creatinine ratio were also associated with kidney disease progression (*p*<0.05 for all). African Caribbean ethnicity was associated with an increased risk of kidney disease progression (HR [95% CI] 1.47 [1.09, 1.98], 1.76 [1.32, 2.36] and 1.57 [1.17, 2.12] for methods 1–3, respectively) and this effect was independent of glycaemic variability and other traditional risk factors.

**Conclusions/interpretation:**

We observed an independent association between HbA_1c_ variability, evaluated using three distinct methods, and significant kidney disease progression in a multi-ethnic type 1 diabetes cohort. Further studies are needed to elucidate the mechanisms that may explain our results and evaluate if HbA_1c_ variability is a modifiable risk factor for preventing diabetic kidney disease progression.

**Graphical Abstract:**

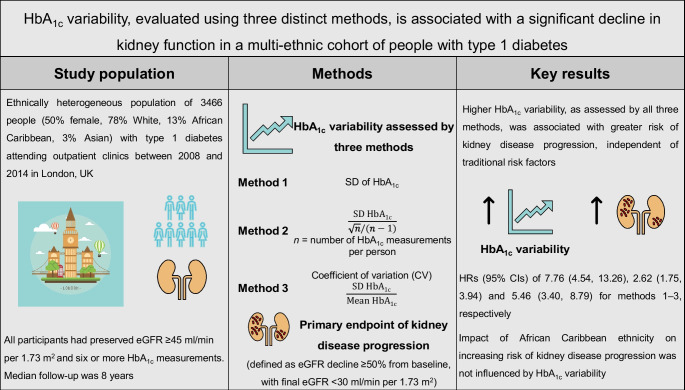

**Supplementary Information:**

The online version contains peer-reviewed but unedited supplementary material available at 10.1007/s00125-024-06197-2.


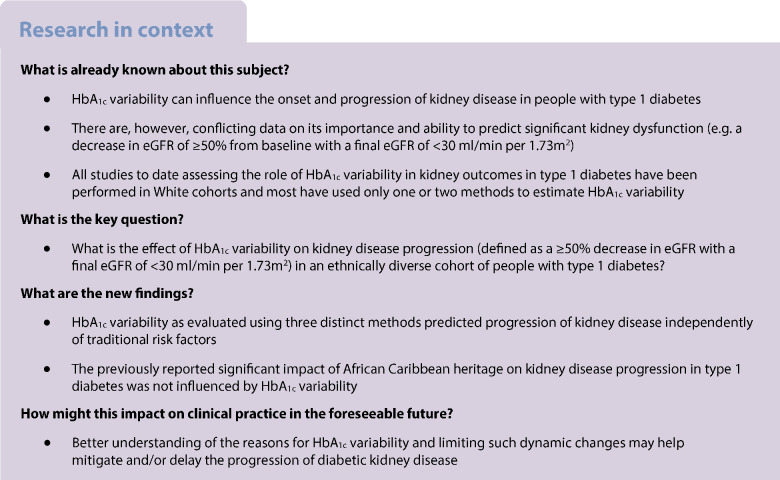


## Introduction

Diabetic kidney disease (DKD) can develop in up to 40% of people with type 1 diabetes and remains a major cause of end-stage kidney failure and premature mortality [1]. Intensive glucose management can prevent the onset and progression of DKD; however, there are conflicting data on the role of HbA_1c_ variability in DKD progression [1, 2]. All studies in this area have been performed in White populations and there remains a lack of knowledge on the role of HbA_1c_ variability in DKD progression in ethnically diverse cohorts of people with type 1 diabetes [3].

Cross-sectional and short-term studies have demonstrated that African American people with type 1 diabetes have higher HbA_1c_ levels and an increased burden of diabetes-related emergency admissions [4]. We previously demonstrated that African Caribbean people with type 1 diabetes show faster DKD progression that is independent of traditional risk factors [5].

In this study we aimed to evaluate if long-term intra-individual HbA_1c_ variability is a risk factor for DKD progression (defined as an eGFR decline of ≥50% from baseline with a final eGFR of <30 ml/min per 1.73 m^2^) in an ethnically heterogeneous cohort of people with type 1 diabetes.

## Methods

Anonymised electronic health record data from people attending routine outpatient care between 2004 and 2018 in two large university hospitals in London (UK) were collected. Full details of the study cohort and the methods used are described elsewhere [5]. Briefly, people with a clinical diagnosis of type 1 diabetes based on primary care, secondary care and/or diabetes eye-screening electronic health records were studied [5]. Information such as date of birth, gender and ethnicity (self-reported), systolic/diastolic blood pressure (SBP/DBP), laboratory measurements such as serum creatinine and urinary albumin/creatinine ratio (ACR), and HbA_1c_ were available.

The primary endpoint was time to DKD progression, defined as an eGFR decline of ≥50% from baseline and a final eGFR of <30 ml/min per 1.73 m^2^. Inclusion criteria included baseline preserved eGFR (defined as ≥45 ml/min per 1.73 m^2^) and six or more HbA_1c_ follow-up measurements. Exclusion criteria were pregnancy, no follow-up eGFR measurement, fewer than six HbA_1c_ measurements or known non-DKD.

Serum creatinine/eGFR and other biochemical/clinical measurements from acute admissions were excluded. People who died during follow-up were excluded from the analyses. The date of the first serum creatinine measurement was the date of entry into the study; HbA_1c_ and all baseline values were extracted within a 2 year timespan and the earliest available data point within this span was reported. Other variables not measured within that timespan were considered missing.

Serum creatinine was used to calculate the eGFR using the Chronic Kidney Disease Epidemiology Collaboration equation [5]. All laboratory tests were performed by the same central provider. Socioeconomic status was measured using the Index of Multiple Deprivation (IMD) and stratified into population deciles, with 1 indicating the highest deprivation level and 10 indicating the lowest deprivation level [5].

Three distinct methods for estimating HbA_1c_ variability were used: (1) SD of HbA_1c_ (SD-HbA_1c_); (2) visit-adjusted HbA_1c_ (adj-HbA_1c_): SD-HbA_1c_/√*n*/(*n*–1), where *n* is the number of HbA_1c_ measurements per participant; and (3) CV (CV-HbA_1c_): SD-HbA_1c_/mean-HbA_1c_ [6–8], stratified into quantiles.

The final follow-up date was the date of DKD progression (if applicable), date of death or date of the last eGFR measurement, whichever was earlier. Multivariate logistic regression models were performed to identify associations between HbA_1c_ variability, estimated using the three distinct methods, and DKD progression, adjusting for clinically relevant variables such as age, gender, IMD deciles, SBP, DBP, log_10_-transformed urinary ACR, ethnicity (stratified into African Caribbean and non-African Caribbean) and baseline HbA_1c_. Continuous variables are presented as mean (SD) or median (IQR) and categorical variables are presented as *n* (%). A *p* value <0.05 was considered significant. All data analyses were performed using RStudio 4.1.1 (R-foundation for Statistical Computing, Vienna, Austria). This retrospective study of anonymised routine clinical data, collected by the direct clinical team, was conducted according to local audit protocols, approved by the hospital data governance committees.

## Results

A total of 3466 people with six or more HbA_1c_ measurements from baseline were analysed, including 1732 (50.0%) women. Participants had a median (IQR) age of 35 (26–46) years. In total, 77.5% of participants were White, 13.2% were African Caribbean, 3.1% were Asian and 6.3% were of unknown ethnicity (defined as of mixed heritage or self-reporting as ‘other’). Overall, this is largely representative of the type-1 diabetes population in England and Wales, as reported in the National Diabetes Audit (NDA) 2019–20, which identified that ~60% of this population were aged between 30 and 59 years, although with more men (57%) [9] than in our study population (50%). Additionally, the NDA 2019–20 reported that 83% of the type 1 diabetes population in England and Wales were White, 3.5% were Asian and 2.3% were African Caribbean [9]. This differs from our study sample; however, this is likely to be due to the location of our hospitals, where African Caribbean people make up ~25% of the local population [10]. Mean (SD) baseline HbA_1c_ was 74.0 (24.5) mmol/mol (8.9% [4.4%]) and eGFR was 91.1 (25.1) ml/min per 1.73 m^2^; median (IQR) urinary ACR was 16.4 (5.5–44.0) mg/mmol (Table 1). Overall, 249 (7.2%) participants progressed to the primary endpoint and 300 (8.7%) participants died within the study period. The median (IQR) follow-up period was 8.2 (4.2–11.6) years.

**Table 1:** Baseline characteristics of participants with type 1 diabetes (*N*=3466)

Characteristic	*N*=3466
Age (years)	35 (26–46)
Gender	
Male	1734 (50.0)
Female	1732 (50.0)
Ethnicity	
African Caribbean	457 (13.2)
Asian	106 (3.1)
White	2686 (77.5)
Mixed heritage or other	217 (6.3)
eGFR (ml/min per 1.73 m^2^)	91.1 (25.1)
Urinary ACR (mg/mmol)	16.4 (5.5–44.0)
Office blood pressure (mmHg)	
SBP	122.9 (15.8)
DBP	73.3 (9.3)
HbA_1c_	
mmol/mol	74.0 (24.5)
%	8.9 (4.4)
IMD decile	3 (2–5)

Continuous variables are presented as mean (SD) or median (IQR) and categorical variables are presented as *n* (%)

Comparison of baseline characteristics identified that African Caribbean participants had significantly higher baseline HbA_1c_ levels and urinary ACR and lower weight and were younger than non-African Caribbean participants, consistent with previous observations [5]. Mean (SD) HbA_1c_ variability estimated using all three methods was significantly higher in African Caribbean participants than non-African Caribbean participants: 13.60 (8.13) vs 8.86 (5.90) mmol/mol for method 1 (SD-HbA_1c_), 0.88 (0.89) vs 0.63 (0.66) mmol/mol for method 2 (adj-HbA_1c_) and 0.18 (0.10) vs 0.13 (0.07) for method 3 (CV-HbA_1c_), respectively (electronic supplementary material [ESM] Table 1). In post hoc analyses comparing people with fewer than six HbA_1c_ measurements (excluded from the primary analysis as recommended [6–8]) with those with six or more HbA_1c_ measurements (who we included), we observed that participants in the former group were younger and had a higher baseline eGFR, DBP and lower urinary ACR, with no other significant differences (ESM Table 2).

Our primary analyses using multivariate Cox regression models identified a significantly higher risk of the primary endpoint of DKD progression with increasing HbA_1c_ variability: compared with those in the lowest HbA_1c_ variability quartile, participants in the highest quartile had HRs (95% CIs) of 7.76 (4.54, 13.26), 2.62 (1.75, 3.94) and 5.46 (3.40, 8.79) using methods 1–3, respectively, independent of risk factors such as age, HbA_1c_, log_10_-transformed ACR and SBP. An increased risk of DKD progression with increasing HbA_1c_ variability (using all methods for estimating HbA_1c_ variability) was observed (Table 2). In our previous work, we observed an enhanced risk of DKD progression in African Caribbean participants compared with non-African Caribbean participants, independent of traditional risk factors [5]; in these additional analyses, this significant effect persisted and was also not influenced by HbA_1c_ variability. No difference in association between HbA_1c_ variability and DKD progression by gender was identified in our regression model (Table 2).

**Table 2:** Impact of HbA_1c_ variability evaluated by three distinct methods and other known traditional risk factors, through multivariate Cox regression modelling, on kidney disease progression in people with type 1 diabetes

Variables	SD-HbA_1c_ (method 1)			Adj-HbA_1c_ (method 2)			CV-HbA_1c_ (method 3)		
	HbA_1c_ variability quartile (mmol/mol)	HR (95% CI)	*p* value	HbA_1c_ variability quartile (mmol/mol)	HR (95% CI)	*p* value	HbA_1c_ variability quartile	HR (95% CI)	*p* value
HbA_1c_ variability	[0.638, 5.22]	Reference	–	[0.0572, 0.261]	Reference	–	[0.0164, 0.0845]	Reference	–
	(5.22, 7.51]	2.00 (1.12, 3.57)	0.019	(0.261, 0.43]	1.77 (1.19, 2.65)	0.005	(0.0845, 0.115]	1.85 (1.10, 3.10)	0.020
	(7.51, 11.6]	3.39 (1.97, 5.55)	< 0.001	(0.43, 0.776]	2.05 (1.39, 3.04)	< 0.001	(0.115, 0.166]	2.46 (1.51, 4.03)	< 0.001
	(11.6, 53.4]	7.76 (4.54, 13.26)	< 0.001	(0.776, 8.55]	2.62 (1.75, 3.94)	< 0.001	(0.166, 0.648]	5.46 (3.40, 8.79)	< 0.001
Age (years)		1.04 (1.04, 1.05)	< 0.001		1.04 (1.03, 1.05)	< 0.001		1.04 (1.03, 1.05)	< 0.001
HbA_1c_ (mmol/mol)		1.01 (1.01, 1.02)	< 0.001		1.02 (1.01, 1.02)	< 0.001		1.02 (1.01, 1.02)	<0.001
SBP (mmHg)		1.01 (1.00, 1.02)	0.014		1.01 (1.00, 1.02)	0.034		1.01 (1.00, 1.02)	0.012
DBP (mmHg)		0.99 (0.98, 1.01)	0.547		1.00 (0.99, 1.02)	0.759		1.00 (0.98, 1.01)	0.712
IMD decile		1.03 (0.98, 1.09)	0.217		1.02 (0.97, 1.08)	0.428		1.03 (0.98, 1.09)	0.267
Urinary ACR		2.03 (1.58, 2.61)	< 0.001		2.33 (1.82, 2.98)	< 0.001		2.14 (1.66, 2.74)	< 0.001
Gender									
Female		Reference	-		Reference	-		Reference	-
Male		0.86 (0.67, 1.11)	0.247		0.89 (0.69, 1.14)	0.349		0.88 (0.68, 1.12)	0.296
Ethnicity									
Non-African Caribbean		Reference	-		Reference	-		Reference	-
African Caribbean		1.47 (1.09, 1.98)	0.011		1.76 (1.32, 2.36)	< 0.001		1.57 (1.17, 2.12)	0.003

## Discussion

We report an independent association between higher HbA_1c_ variability and DKD progression, defined as a ≥50% eGFR decline from baseline with a final eGFR of <30 ml/min per 1.73 m^2^, in an ethnically heterogeneous cohort of people with type 1 diabetes, independent of traditional risk factors associated with DKD progression.

These results are consistent with data from White cohorts, for which an association between the HbA_1c_ CV and microvascular disease has been observed, albeit in a smaller sample (*n*=1240) [11]. A meta-analysis of four studies (three from Europe and one from North America) showed that higher HbA_1c_ variability assessed using a single method was associated with poor kidney outcomes [12]. Our cohort is the most ethnically diverse studied to date (23% of participants were of African Caribbean, Asian or unknown ethnicity) and we observed similar results using three different methods of estimating HbA_1c_ variability. There was an equal distribution of men and women (50% for each) within the study population, and gender did not appear to play a role in the association of glycaemic variability and DKD progression.

The strengths of our study include its contemporaneous nature, long median follow-up time of 8.2 years and the use of the three distinct methods to estimate HbA_1c_ variability, ensuring that the analysis was robust. Adj-HbA_1c_ (method 2) was used as participants do not necessarily have a standard time gap between visits and hence the regularity of clinic visits may differ. Notably, we observed a consistent, independent effect of HbA_1c_ variability on DKD progression for all three methods.

Our results demonstrate that relying only on mean HbA_1c_ levels may mask the impact of underlying variable (‘erratic’) HbA_1c_ history; even after adjusting for known clinical risk factors for DKD progression, HbA_1c_ variability (assessed using all three methods) remained an independent significant predictor of DKD progression.

HbA_1c_ variability may be retained as an adverse ‘metabolic memory’ due to consequent epigenetic changes sustained from significant episodes of hyperglycaemia, which can damage the microvasculature [13]. A recent study in people with type 1 diabetes suggested that within-day 7 point capillary glucose measurement variability was not associated with DKD onset [14]. In contrast, glycaemic optimisation using sensor-augmented insulin pump therapy and continuous glucose monitoring (CGM) systems reduced glycaemic variability and improved time in range, which was associated with a reduction in albuminuria in people with type 1 diabetes and DKD [15].

There are several limitations to our study. Our study cohort was based on two large urban university hospitals where people with advanced diabetes/needing challenging diabetes care are referred, and this may explain the high baseline HbA_1c_ observations. These hospitals are part of a publicly funded healthcare system and hence our results may be less applicable to other healthcare systems. There were no available data on medical therapy or its history, which may have had an impact on our results. There are conflicting data on the role of race and ethnicity in the use of renin–angiotensin system inhibitor, which are recommended for preventing DKD progression; some studies suggest lower use/prescription rates in African Caribbean populations, whereas others do not [16, 17]. Differences in healthcare systems, populations and study methods may explain these discrepancies.

Participants with fewer than six HbA_1c_ measurements post baseline were excluded (to ensure robustness of the methods used to estimate HbA_1c_ variability) and this may have resulted in selection bias. Time-weighted mean HbA_1c_ was not adjusted for in this study, as our aim was to understand the role of HbA_1c_ variability. Similarly, the variability of other risk factors, such as blood pressure, which may affect kidney outcomes [18], was not explored. Further studies are needed to investigate the variability of multiple risk factors in DKD progression. Type 1 diabetes diagnosis was based on medical/eye-screening records and it is possible that participants with ketosis-prone type-2 diabetes may have been mislabelled as having type 1 diabetes; however, a comparison of participants with African Caribbean and non-African Caribbean ethnicity did not demonstrate a higher BMI/weight or older age in the former, which may be prevalent in the ketosis-prone type 2 diabetes phenotype [5]. In our study, socioeconomic deprivation, measured using nationally approved methods, was not associated with the endpoint of progression of DKD; however, we acknowledge that more nuanced socioeconomic/healthcare indices are needed to fully assess the impact of socioeconomic factors on DKD outcomes. Finally, our retrospective study design cannot prove a causal relationship between HbA_1c_ variability and DKD progression.

### Conclusions

In an ethnically diverse type 1 diabetes cohort we observed an association between HbA_1c_ variability, evaluated using three distinct methods, and clinically significant DKD progression, defined as an eGFR decline of ≥50% and a final eGFR of <30 ml/min per 1.73 m^2^. Our result reinforces the role of optimal stable HbA_1c_ in preventing DKD progression. Further research is required to evaluate ‘short-term’ glycaemic variability (e.g. from CGM data), as this may help to further elucidate the effect of glycaemic variability on DKD and microvascular complications.

## Supplementary Information

Below is the link to the electronic supplementary material.

Supplementary file1 (PDF 766 KB)

## Data Availability

The data that support the findings of this study are not openly available for reasons of participant confidentiality and are available from the corresponding author on reasonable request.

## Abbreviations

ACR: Albumin/creatinine ratio
CGM: Continuous glucose monitoring
DBP: Diastolic blood pressure
DKD: Diabetic kidney disease
IMD: Index of Multiple Deprivation
NDA: National Diabetes Audit
SBP: Systolic blood pressure
