# Supplementary material for: HbA1c variability is independently associated with progression of diabetic kidney disease in an urban multi-ethnic cohort of people with type 1 diabetes
Source: Diabetologia. 2024 Jun 20;67(9):1955–61. doi: 10.1007/s00125-024-06197-2 (PMC11410835; doi:10.1007/s00125-024-06197-2)
Supplement: Supplementary file 1 — Supplementary file1 (PDF 766 KB) [file 125_2024_6197_MOESM1_ESM.pdf]

Electronic Supplementary Materials

ESM Table 1: Baseline characteristics and HbA_{1c} variability as assessed by three distinct methods in people with type-1 diabetes of non-African-Caribbean [White, Asian, Unknown (mixed-heritage or other)] and African-Caribbean origin.

		Non-African Caribbean (N = 3009)	African Caribbean (N = 457)	P-value
**Age (Years)***		35 (27-46)	32 (19-42)	<0.001
**Sex N (%)**				
	Male	1515 (50.4)	219 (48.2)	0.3857
	Female	1494 (49.6)	238 (51.8)	
**Weight (kg)**		72.6 (17.2)	69.5 (21.1)	<0.001
**BMI (kg/m²)**		25.2 (4.8)	25.1 (5.9)	0.213
**Albumin : Creatinine ratio (ACR) (mg/mmol)***		14.6 (5.3 – 43)	36.5 (7.2 - 45)	<0.001
**Office Blood Pressure (mmHg)**				
	Systolic blood pressure	123 (15.7)	122 (16.8)	0.456
	Diastolic blood pressure	73.3 (9.2)	73.5 (10.3)	0.910
**HbA1c**		72.1 (23.0) mmol/mol	86.4 (29.7) mmol/mol	<0.001
		8.7 (4.3) %	10.1 (4.9) %	
**HbA1c Variability Methods**				
	**Method 1** $(Standard\ deviation\ of\ HbA1c)$	8.86 (5.90)	13.60 (8.13)	<0.001
	**Method 2** $\frac{Standard\ deviation\ (HbA1c)}{\sqrt{[n/(n-1)]}}$	0.63 (0.66)	0.88 (0.89)	<0.001

**Method 3** $\frac{Standard\ deviation\ (HbA1c)}{Mean\ (HbA1c)}$	0.13 (0.07)	0.18 (0.10)	<0.001
**eGFR (mL/min per 1.73m^2)**	91.3 (24.4)	90.7 (29.2)	0.154
**Indices of multiple deprivation (IMD) Decile***	3 (2-5)	3 (2-5)	0.337

ESM Table 1: Continuous and categorical variables analysed through a Mann-Whitney-U Test and a Chi-Square Test, respectively. Data represented as mean (SD), median (IQR)*, or N (%). Post-hoc analyses, so p-values are to be interpreted with caution as exploratory results.

ESM Table 2: Comparison of baseline characteristics of participants with <6 HbA_{1c} measurements (who were excluded from analyses) with those with ≥6 HbA_{1c} measures (who were analysed/studied)

	<6 HbA1c measurements N = 805	≥ 6 HbA1c measurements N = 3466	P-value
**Age (Years)***	32 (25 – 42)	35 (26 – 46)	<0.001
**Ethnicity N (%)**			
Non-African Caribbean	688 (85.5)	3009 (86.8)	0.3015
African-Caribbean	117 (14.5)	457 (13.2)	
**Sex N (%)**			
Male	377 (46.8)	1734 (50.0)	0.102
Female	428 (53.2)	1732 (50.0)	
**Weight (kg)**	71.9 (17.9)	72.2 (17.8)	0.945
**eGFR (mL/min per 1.73m^2)**	98.0 (25.8)	91.1 (25.1)	<0.001
**BMI (kg/m^2)**	24.8 (4.7)	25.2 (4.9)	0.1205
**Office Blood Pressure (mmHg)**			
Systolic blood pressure	123.0 (14.8)	122.9 (15.8)	0.8
Diastolic blood pressure	74.1 (9.0)	73.3 (9.3)	0.028
**Urine Albumin : Creatinine Ratio (ACR) (mg/mol) ***	11.8 (5.2 – 43.0)	16.4 (5.5 – 44.0)	0.037
**Baseline HbA_{1c}**	75.6 (26.8) mmol/mol	74.0 (24.5) mmol/mol	0.315
	9.1 (4.6) %	8.9 (4.4) %	
**Indices of multiple deprivation (IMD) Decile***	3 (2-5)	3 (2-5)	0.777

ESM Table 2: Continuous and categorical variables analysed through a Mann-Whitney-U Test and a Chi-Square Test, respectively. Data represented as mean (SD), median (IQR)*, N (%). Post-hoc analyses so p-values are to be interpreted with caution as exploratory results.
